# A Small Network MicronNet-BF of Traffic Sign Classification

**DOI:** 10.1155/2022/3995209

**Published:** 2022-03-18

**Authors:** Hai-Feng Fang, Jin Cao, Zhi-Yuan Li

**Affiliations:** ^1^School of Mechanical Engineering, Jiangsu University of Science and Technology, Zhenjiang 212100, China; ^2^College of Engineering and Computer Science, Australia National University, Canberra, Australian Capital Territory 2601, Australia

## Abstract

One of a very significant computer vision task in many real-world applications is traffic sign recognition. With the development of deep neural networks, state-of-art performance traffic sign recognition has been provided in recent five years. Getting very high accuracy in object classification is not a dream any more. However, one of the key challenges is becoming making the deep neural network suitable for an embedded system. As a result, a small neural network with as less parameters as possible and high accuracy needs to be explored. In this paper, the MicronNet which is a small but powerful convolutional neural network is improved by batch normalization and factorization, and the proposed MicronNet-BN-Factorization (MicronNet-BF) takes advantages about reducing parameters and improving accuracy. The effect of image brightness is reduced for feature recognition by the elimination of mean and variance of each input layer in MicronNet via BN. A lower number of parameters are realized with the replacement of convolutional layers in MicronNet, which is the inspiration of factorization. In addition, data augmentation is also been changed to get higher accuracy. Most important, the experiment shows that the accuracy of MicronNet-BF is 99.383% on German traffic sign recognition benchmark (GTSRB) which is much higher than the original MicronNet (98.9%), and the most influence factor is batch normalization after the confirmation of orthogonal experimental. Furthermore, the handsome training efficiency and generality of MicronNet-BF indicate the wide application in embedded scenarios.

## 1. Introduction

**Figure 1:**
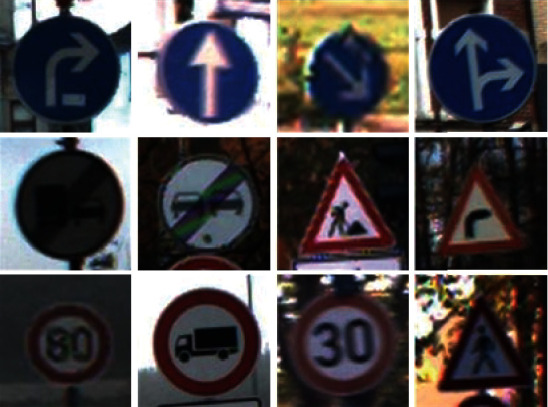
Various categories of traffic signs under different weather and brightness in GTSRB.

Traffic signs, usually erected at the side of roads, use texts or symbols to provide road information for vehicle divers and pedestrians (see Figure 1). Traffic sign recognition is essential in advanced driver assistance systems (ADASs) and autonomous vehicles [1]. In the real cases, camera installed on the vehicle takes photos of roads. The information processing system processes the image and detect and classify the traffic sign according to its characters. The classification result provides road information for drivers or adjusts the motion state of an autonomous vehicle. Because the captured images are affected by brightness and weather conditions, traffic sign classification has high requirement in accuracy and robustness.

For the sufficient research of traffic sign recognition, researchers established a multitraffic sign recognition dataset such as German Traffic Sign Recognition Benchmark (GTSRB) [2], Belgium Traffic Sign Classification Benchmark (BelgiumTSC) [3], and Tsinghua-Tencent 100K dataset [4]. GTSRB dataset provides 51,840 colorful images of German road signs in 43 classes. This dataset also provides cropped images for accurate classification. Most of images are clear, but part of them is blurred and darkness used to test the algorithm's robustness. It not only allows researchers to test the accuracy of their algorithm and to compare it with human performance but also to be transformed by the histogram of the oriented gradient algorithm to prevent projection distortion [5] or denoised to promise the quality of dataset [6].

In recent years, convolution neural networks (CNNs) show high performance in the GTSRB dataset [7–9]. CNNs, inspired from human's visual perception mechanism, are applied broadly in computer vision [10]. As a deep learning network, it has many layers to simulate neurons to learn the characters of images. It has showed high performance in many datasets such as CIFAR [11] and ImageNet [12], so people consider applying the enhanced CNN (e.g., LeNet-5 [13], Caps Net [5], PFANet [14], differential evolution evolved RBFNN [15], etc.) in traffic classification. However, application in vehicles has its restriction. The network requires high response speed under the limited storage space. The hardware installed on the vehicle does not have enough computation ability, which causes the scale of the network limited [16]. As such, some famous mature deep networks such as GoogLeNet [17] and VGG [18] are too deep or huge to be applied in vehicles directly. However, small networks are feasible. Zhang et al. [1] proposed two light weight CNN simple student network and deep teacher network and assisted the training of the student model to achieve high accuracy in traffic sign classification. Arman et al. [19] proposed a novel thin yet deep convolutional neural network for a light weight architecture. Cao et al. [20] used HSV color space preprocess images and applied improved LeNet-5 CNN model with a small number of parameters in traffic sign classification. Although the research studies above have slimmed the networks to adapt the embedded system, the recognition of brightness and blur pictures of traffic sign is still an arduous challenge. Wong et al. [21] proposed MicronNet and trained it with the augmented (e.g., HSV augmentation, Gaussian blur, motion blur, etc.) traffic sign photos. However, the augmented dataset in [21] have no emphasis, causing information redundancy. There are still a number of optimizations in the structure of MicronNet and the augmentation of traffic sign dataset, and it is essential to find a proper balance between the processing of a traffic sign dataset and the light weight structure of the convolutional neural network.

Inspired from the above network, we proposed a CNN based on MicronNet, a small network and overcomes drawbacks of the original network. In this study, we mainly focus on MicronNet-BN-Factorization (MicronNet-BF) which fused the superiority of MicronNet, batch normalization, and factorization. In addition, the appropriate augmentation methods of insufficient illumination traffic signs are selected for a better training performance.

The main contributions of this paper can be summarized as follows:

  The complicated data augmentation methods (including shift, flip, mirror, HSV, blur, and rotation) are simplified into shift, scale, and the V channel of HSV, avoiding that too much data augmentation may introduce some useless or even false characters of traffic sign and reduce the accuracy of the neural network.
  Two channels are additionally supplemented to the first layer, 1-by-1 convolution, to enhance the learning of image features from the dataset. 5-by-5 convolutional layer is replaced by two sequential 3-by-3 convolutional layers, reducing parameters and extracting more meticulous characteristics to increase the accuracy.
  Batch normalization can learn and fix the input means and variances of each layer. For the traffic sign recognition, the adverse effect of brightness is effectively reduced, improving the classification accuracy of insufficient lighting images.

## 2. Related Work

**Figure 2:**
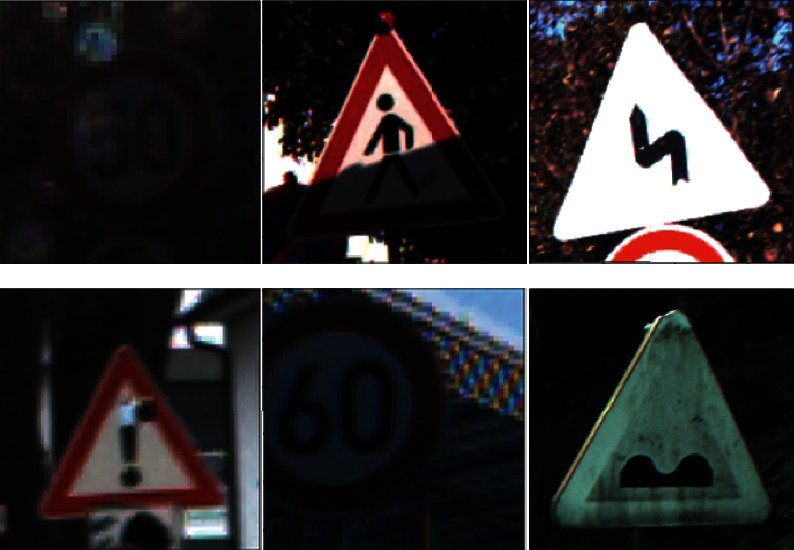
Examples of traffic images in GTSRB unrecognized by original MicronNet since blur or darkness.

MicronNet [21], a small deep convolutional neural network, is proposed to achieve real-time embedded traffic sign classification. The network structure is optimized from a large network by repeating omitting parameters and testing network to maintain high accuracy with the least number of useful parameters. The final optimized network reaches 98.9% accuracy only containing 0.51 M parameters and which is competitively with the deep inception-based CNN [22] with 10.5 M or single CNN with 3 STNs [23] with 14 M, etc. Furthermore, a few logical operations are required for MicronNet to perform inferences and short computation time meanwhile. However, the network cannot deal with dark and blurred images well (see Figure 2). Based on the MicronNet, we adjust data augmentation and modify parts of the network to make it suitable to both common images and dark images.

Ioffe and Szegedy [24] proposed batch normalization (BN) to improve classification accuracy and training rate. Because of internal covariate shift, the changed parameters of the previous layers causing each layer inputs changed after every training epoque, and traditional network training chooses a low learning rate. Batch normalization normalize every layer's input for each training minibatch. We introduce batch normalization to MicronNet to improve its learning rate and accuracy.

Szegedy et al. [25] based on GoogLeNet [17] presented the inception V2 network. In this paper, Szegedy presented a theory that two sequential small convolutional filters can replace a large convolutional filter to improve the learning rate and reduce parameter number and achieve similar accuracy because the receptive field of two methods are the same. The factorizing mentality is fused in MicronNet either.

## 3. Proposed Methods

### 3.1. Data Augmentation

**Figure 3:**
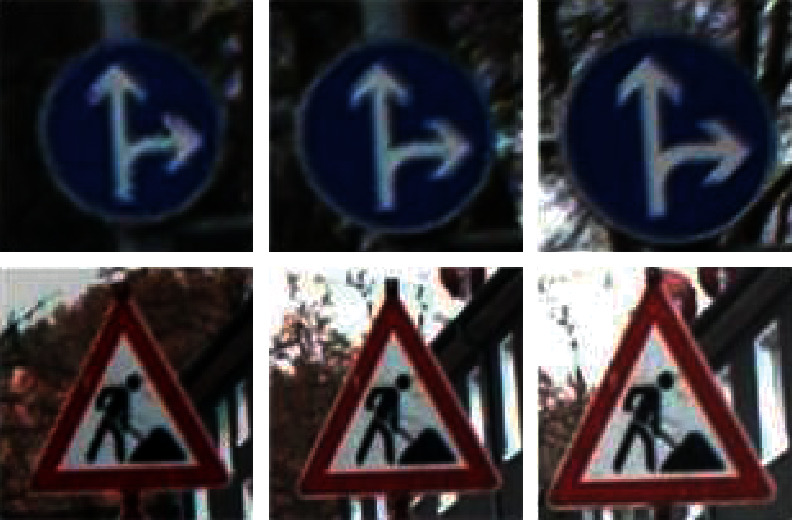
Data augmentation of traffic signs in GTSRB. The data augmentation ways about shifting, brightness and scale can be seen from images.

The uneven distribution of data will be decreasing the accuracy of classification. Researchers use a various of data augmentation techniques to balance the number of samples [21, 26]. However, on the one hand, the data that can be augmented based on one sample is limited and cannot be increased indefinitely due to the distortion of the characteristics of sample in the process of augmentation. On the other hand, the ministructure of the neural network cannot effectively learn too much characteristics. As a result, the proposed data augmentation is simplified to three ways: (i) shifting, (ii) brightness, and (iii) scale. The properties of choosing these three ways can be described as follows: (1) Shifting can help to deal with the partially covered traffic signs. (2) Brightness can help to learn the traffic signs under different light conditions. (3) Scale can help to handle various sizes of traffic signs. The examples can be seen in Figure 3.

### 3.2. MicronNet-BF

MicronNet is a compact deep neural network proposed for traffic sign classification on embedded devices [21]. It has struck a relative balance between the augmentation of a traffic sign dataset and the simplifying of the network architecture, but the main problem in the example of misclassified traffic images is either heavily motion blurred (left), partially occluded (middle), or exhibit poor illumination (right). Based on the MicronNet and inspired from the network architecture of inception V2 [21, 25], an improved network architecture MicronNet-BN-factorization (MicronNet-BF) is proposed in this paper. MicronNet-BF is taken to (1) improve the total accuracy on traffic sign recognition problems, (2) keep the same model size or achieve a smaller model size for embedded devices, (3) achieve better performance on classification accuracy of a special class (low brightness images).

**Figure 4:**
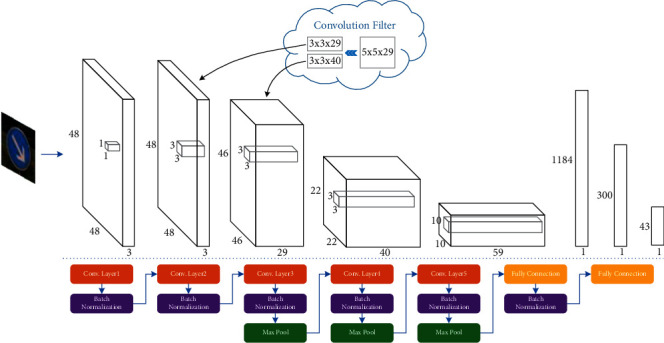
Brief structure diagram of MicronNet-BF. The sizes of convolution filters and convolution layer's inputs are expressed on the upper diagram, and the brief description of MicronNet-BF's complete layer structure on the below.

Figure 4 shows the overall network architecture of MicronNet-BF, and Table 1 prints the details of parameters. In this architecture, it mainly has 5 convolutional layers, 2 fully-connected layers, and a SoftMax layer. All the activation functions in this network are chosen to be rectified linear unit (ReLU) for the reducing of computational complexity. In this network, the 1-by-1 convolutional layer in the original MicronNet is extended to have 3 output channels, and the 5-by-5 convolutional is replaced by two of the 3-by-3 layers. Furthermore, batch normalization layers are added into the proposed network to deal with the brightness difference in the input images and improve training speed.

The batch normalization layer in a network learns the mean and variance of dataset, and fixes the input means and variances of each layer. In the application of traffic sign classification, the brightness of each input image is closely related to the mean and variance value of the image. By normalization of the mean and variance of each image, the batch normalization layer turns all the images in the dataset to have a similar brightness, which improves the classification accuracy of the low brightness traffic sign images.

On the one hand, inspired by the idea of “factorization” into smaller convolutions in inception V2 [25], the 5-by-5 convolutional layer is replaced by two of the 3-by-3 convolutional layers, as shown in Table 1. The 3-by-3 convolutional layer used in this replacement enables the network to learn some smaller scaled feature from the input images and share the features among the following up 3-by-3 convolutional layer. Furthermore, the spatial coverage of the original 5-by-5 layer is maintained by the overlap of two 3-by-3 convolutional layers. In this way, this improvement results in a slight deeper network with the ability of learning smaller scaled details from the traffic signs, which significantly improved the overall classification accuracy.

On the other hand, traffic signs are normally designed with colors of high contrast, including black, white, red, yellow, and blue. In order to use the color information in the traffic sign classification, the 1-by-1 convolutional layer is extended to have 3 output channels. In the traditional network, the 1-by-1 convolutional layer combines the RGB color of the input image to 1 value on each pixel location, which can be considered as a RGB to gray conversion. After extending the output channel to 3, the 1-by-1 convolutional layer becomes a color extraction layer, which provides 3 different color combinations for the following up layers.

## 4. Experimental Evaluation

### 4.1. Implementation Details

For a fair comparison, both methods and algorithm architectures take similar learning hyperparameters. A learning rate of 0.007 is used for MicronNet and MicronNet-BF in most scenes. The hyperparameters of comparison methods are taken as the default of the corresponding cite paper. Meanwhile, the same experiment platform is used in all experiment and is a Linux Ubuntu20.4 operating system with PyTorch. The GPU is GTX1080ti of NVIDIA. As an evaluation index, the accuracy rate refers to the percentage of the number of correctly recognized in the test dataset to the whole number of test dataset, and time refers to the sum of training time of 100 samples in each epoch. Additionally, some abbreviations for networks have been adopted for briefly expression, as shown in Table 2.

**Figure 5:**
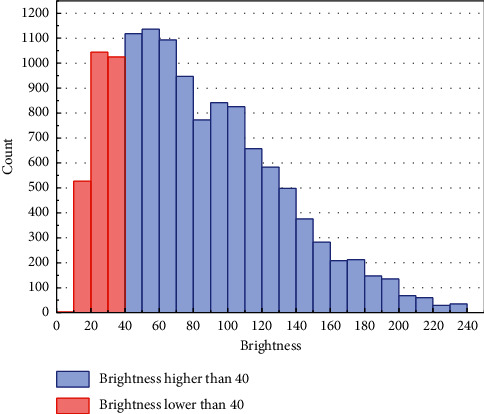
Testing set brightness distribution. The samples with brightness lower than 40 are constituted to the brightness insufficient dataset.

For the comparing of the recognition of networks on dark images, the insufficient brightness images of traffic sign are extracted from the testing dataset to combine a new challenging dataset. After ordering the brightness of the whole testing dataset, the first 20.57% samples (the number of 2599) were used as the new insufficient illuminated traffic sign dataset; that is, the average brightness of each sample in the new dataset was lower than 40. The quantity distribution of the testing dataset is shown in Figure 5. The samples with red are constructed to a harder dataset, and the rest samples are used for testing.

### 4.2. MicronNet-BF Evaluation with GTSRB

For the comparing with MicronNet [21], the proposed MicronNet-BF is evaluated on German traffic sign recognition benchmark (GTSRB) [27] firstly. The GTSRB dataset contains color traffic sign images from 43 classes and intends for recognition. On the one hand, the evaluation with the overall accuracy on GTSRB is taken normally. For further challenges, the recognition of lower brightness images from the GTSRB testing set is processed meanwhile. On the other hand, during the training of the network, rotation, shifting, and scaling are used as data argumentation strategies to improve the generality of the resulting network, especially for the testing images with partly visible sign.

**Figure 6:**
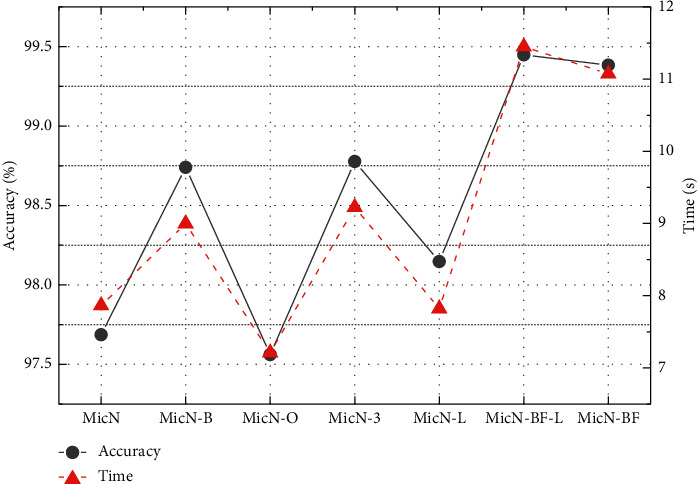
Accuracies and time of networks based on GTSRB. The best accuracy with 99.448% is taken with MicronNet-BF under the insufficient brightness dataset.

Figure 6 shows the testing accuracy and training time of the proposed network MicronNet and the comparison networks based on GTSRB dataset. Comparing with the MicronNet, the batch normalization layer added into it improves the classification accuracy from 97.686% to 98.74%. Furthermore, the extending of output channels on 1-by-1 layer improves the accuracy to 97.561%, and the replacement of two 3-by-3 layers improves the overall accuracy to 98.777%. Thus, the overall accuracy with 99.383% of the MicronNet-BF is improved by the three strategies proposed in this project, respectively. What is more, the comparison of MicN-BF × L with 99.448% and MicN-L with 98.147% indicates the great recognition performance of MicronNet-BF under insufficient brightness.

### 4.3. Validation of MicronNet-BF Influence Factors

In the front section experiment, it was proved that batch normalization, extending of output channels on 1-by-1 layer, and factorization were successfully integrated into MicronNet, but the effect and influence processes of each factor need further experimental verification. The Taguchi orthogonal array experimental method can greatly reduce the number of experiments than grid searching experiment and inference of the optimal parameter combination by the orthogonal method [28, 29]. The Taguchi orthogonal array experimental method is used to obtain the optimal values and evaluate the influence of factors [30, 31].

There are three factors in MicronNet-BF that need to be focused on. In addition, the interaction between factors should also be considered, including dataset with insufficient illumination. For a summary, there are seven factors (including MicN-B, MicN-O, MicN-3, MicN-L, MicN-B × O, MicN-B × 3, and MicN-O × 3) and two levels (including N (NULL) and A (APPLY)) in the experiment. With respect to selected factors, *L*_8_(2^7^) orthogonal array was designed for the validation experiments, and the experimental design and the results are shown in Table 3.

For an immediate point of view, the best accuracy with 99.448% is taken with the network of MicronNet-BF under the insufficient brightness testing dataset; it is consistent with the conclusion of the previous subsection. In Table 3, *I*_*A*1_ denotes the summary of accuracy under the first level of the factors, and *I*_*A*2_ for the second level, *R*_*A*_ represents the absolute value of the difference of *I*_*A*1_ and *I*_*A*2_. The meaning of *I*_*T*1_, *I*_*T*2_, and *R*_*T*_ is similar with the third before but for time. From the row of *R*_*A*_, the biggest difference is the factor of MicN-B and the smallest is MicN-O, and it indicates that the factor MicN-B has the most influence of the recognition accuracy of traffic signs, and the factor MicN-O has the lowest influence. The result in the row of RT shows that the factor MicN-B has the most influence of the training time too, but the factor MicN-B *X* O has the lowest. According to the difference value of interaction factor, there is only a tiny effect about accuracy and time. Therefore, the ranking of effects can be sorted as MicN-B > MicN-3 > MicN-L > MicN-B × 3 > MicN-O × 3 > MicN-B *X* O > MicN-O.

**Figure 7:**
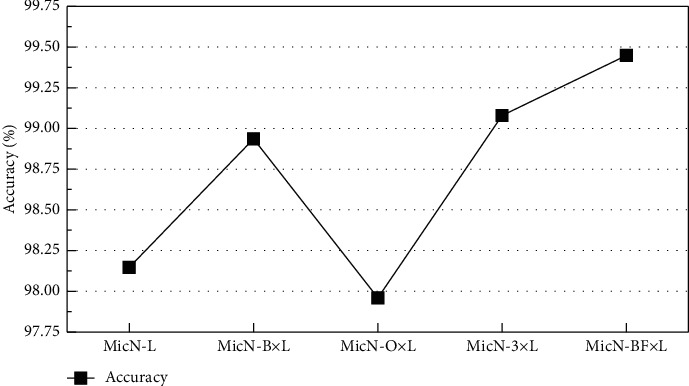
Accuracies of networks based on insufficient brightness data. The best accuracy with 99.448% is taken by MicronNet-BF.

For the insufficient brightness traffic sign dataset, the recognition accuracy is shown in Figure 7. The best accuracy with 99.448% is taken by MicronNet-BF, and the accuracies of MicN-B × L with 98.936% and MicN-3 × L with 99.079% are better than MicronNet significantly. The recognition ability of batch normalization and factorization for traffic signs with insufficient brightness is proved. Although the accuracy of MicN-O × L with 97.96% is no better than others, this tendency can be seen in Table 4 with the *R*_*A*_ of MicN-O. On the one hand, it is indicated that extending the output of the 1 by 1 layer to three channels cannot enhance the recognition performance of traffic signs with insufficient brightness, but MicN-O can improve the classification performance of traffic signs with normal illumination and rich colors to a certain extent, and hardly increase the extra training time meanwhile. Therefore, the MicN-O is also effective.

**Figure 8:**
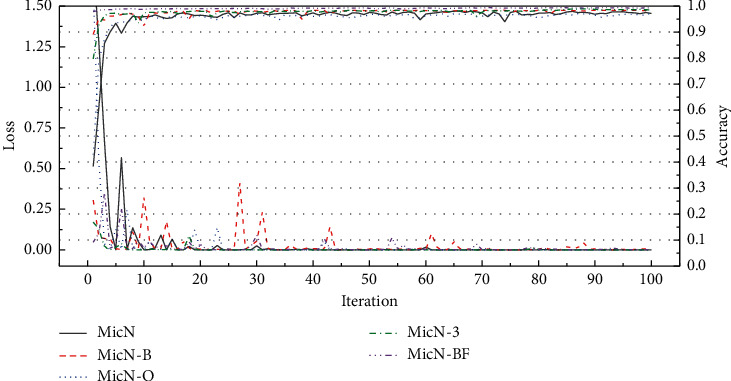
The accuracies and losses of networks. Obviously, the loss value of MicN-B is more volatile.

On the other hand, the fluctuation of loss value and accuracy rate in the process of iteration can also reflect the role of various factors. As Figure 8 shows, networks present various fluctuation trends in the training process. MicN fluctuated widely in the first 10 iterations and remained fairly flat thereafter. The loss value of MicN-B dropped quickly, but the subsequent fluctuations lasted for a long time. The loss value of MicN-O dropped faster than MicN and have a bit fluctuation later. MicN-3 get the best performance in the process of iteration, dropped fastest, and more flatted. Finally, under the balance of various factors, MicN-BF loss value decreases rapidly with few fluctuations so as to achieve the best classification performance quickly and maintain stability.

### 4.4. Comparison Evaluation

With the discussion in the previous subsections, the test of MicronNet-BF on GTSRB dataset is quite complete. In order to further verify the recognition performance of MicronNet-BF on addition traffic sign dataset and different types of datasets, some representative datasets were selected. The properties of several dataset and the evaluation performance are listed in Table 4.

The Belgium Traffic Signs Classification dataset has 62 categories, 4,591 samples for training and 2498 for testing. The results show that the recognition performance of MicronNet-BF with 82.122% is better than MicronNet with 80.388%. It indicated that with the training of a small number of traffic signs, the classification performance of MicronNet-BF decreased, but it was still higher than MicronNet. On the one hand, the generalization of MicronNet-BF and MicronNet has been verified with the accuracies of 99.58% and 99.49% on the dataset of MNIST. In the more challenging number classification dataset of SVHN, MicronNet-BF maintained a slight advantage, indicating that the structural superiority is not limited to the recognition of traffic signs. In the case of more complex dataset Cifar10 and Cifar100, MicronNet-BF is unable to learn deeper features as to its lightweight structure, and the recognition accuracy is only 78.67% and 49.93%, respectively, but it still far exceeds MicronNet with 34.83% and 10.33%.

On the other hand, the MicronNet-BF is mainly used in embedded devices. By comparing the difference of structure between MicronNet-BF and MicronNet, replacing a 5-by-5 convolution filter with two 3-by-3 convolution filters has the greatest impact on the number of variables, and the number of variables is reduced to 0.44 M. As listed in Table 5, compared with the state-of-art networks, with the minimum number of variables of 0.44 M, MicronNet-BF has achieved excellent results with a difference of no more than 0.4% compared with the larger networks.

## 5. Conclusions

In order to improve the recognition performance of traffic signs further, the MicronNet-BF which is fused by MicronNet, batch normalization, and factorization is proposed. The addition of batch normalization enhances the recognition performance to 98.74%, which is 1.05% higher than the performance of MicronNet. The application of factorization improves the accuracy to 98.77%. The MicronNet-BF which is combined by multifactors listed above has a recognition performance with 99.383% which has a great improvement than MicronNet. On the one hand, the batch normalization and factorization do enhance the ability of recognizing the traffic signs with insufficient brightness after the experiment evaluation. On the other hand, the most influence factor is batch normalization after the confirmation of orthogonal experimental. In the end, the performance of MicronNet-BF used in BTSC, Cifar10, and Cifar100 is better than MicronNet.

Although the algorithm is applied in the embedded system, the less parameters are not better, and striking a balance between the number of parameters and the size of storage space needs further study.

## Figures and Tables

**Table 1:** The structure of proposed MicronNet-BF.

Type/stride/pad	Filter shape	Input size
Conv/s1/p0	1 × 1 × 3	48 × 48
BN	Batch normalization	48 × 48
Conv/s1/p0	**3** × **3** × **29**	48 × 48
BN	Batch normalization	46 × 46
Conv/s1/p0	**3** × **3** × **40**	46 × 46
BN	Batch normalization	44 × 44
Pool/s2/p0	3 × 3	Maxpool
Conv/s1/p0	3 × 3 × 59	22 × 22
BN	Batch normalization	22 × 22
Pool/s2/p0	3 × 3	Maxpool
Conv/s1/p0	3 × 3 × 74	10 × 10
BN	Batch normalization	10 × 10
Pool/s2/p0	3 × 3	Maxpool
FC/s1	1 × 300	1 × 1184
BN	Batch normalization	1 × 300
FC/s1	1 × 300	1 × 300
Softmax/s1	Classifier	1 × 43

The bold numbers mean one of the innovations in applying inception V2 to MicronNet.

**Table 2:** Abbreviations of networks.

Abbreviation	Network
MicN	MicronNet (self-trained)
MicN-B	MicronNet + BN
MicN-O	MicronNet + 1-by-1 × 3 outputs
MicN-3	MicronNet + 2 × 3-by-3
MicN-L	MicronNet + insufficient lighting
MicN-BF	MicronNet-BN-factorization (the fusion of all above)

**Table 3:** Orthogonal experimental design and the results.

Ex. No.	MicN-B	MicN-O	MicN-B × O	MicN-3	MicN-B × 3	MicN-O × 3	MicN-L	Acc (%)	Time (s)
1	N	N	N	N	N	N	N	97.686	**7.863**
2	N	N	N	A	A	A	A	99.079	9.564
3	N	A	A	N	N	A	A	97.96	8.371
4	N	A	A	A	A	N	N	98.534	8.858
5	A	N	A	N	A	N	A	98.936	9.708
6	A	N	A	A	N	A	N	99.28	11.707
7	A	A	N	N	A	A	N	98.824	9.509
8	A	A	N	A	N	N	A	**99.448**	11.448
*I*_*A1*_	393.259	394.981	395.037	393.406	394.374	394.604	394.324		
*I*_*A2*_	396.488	394.766	394.71	396.341	395.373	395.143	395.423		
*R*_*A*_	**3.229**	**0.215**	0.327	2.935	0.999	0.539	1.099		
*I*_*T1*_	34.656	38.842	38.384	35.451	39.389	37.877	37.937		
*I*_*T2*_	42.372	38.186	38.644	41.577	37.639	39.151	39.091		
*R*_*T*_	**7.716**	0.656	**0.26**	6.126	1.75	1.274	1.154		

The bold numbers represent the best performance of the corresponding item.

**Table 4:** The testing accuracy and training time.

	Dataset	GTSRB	BTSC [3]	MNIST	SVHN [32]	Cifar10	Cifar100
Property	Categories	43	62	10	10	10	100
	Training samples	39209	4591	60000	73257	50000	50000
	Testing samples	12630	2498	10000	26032	10000	10000

MicronNet	Testing accuracy	97.686%	80.388%	99.49%	92.16%	34.829%	10.333%
	Training time	1 s	1.01 s	0.55 s	0.38 s	0.85 s	0.85 s

MicronNet-BF	Testing accuracy	**99.383%**	**82.122%**	**99.58%**	**93.35%**	**78.67%**	**49.93%**
	Training time	1.41 s	1.48 s	0.60 s	0.65 s	1.29 s	1.27 s

The bold numbers represent the one with the better test accuracy in MicronNet-BF and MicronNet.

**Table 5:** Classification accuracies of GTSRB and parameter number of algorithms.

Network	Parameter (M)	Accuracy (%)
Deep inception-based CNN [24]	10.5	99.81
Single CNN with 3 STNs [30]	14	99.71
HLSGD [33]	23.2	99.65
Student network [1]	0.73	99.61
MCDNN [16]	38.5	99.46
**MicronNet-BF**	**0.44**	**99.38**
MicronNet [21]	0.55	98.9
DNN [16]	1.54	98.52

The bold characters and numbers represent the proposed algorithm and its parameters, respectively.

## Data Availability

All the dataset could be found in https://paperswithcode.com/datasets, including GTSRB, BTSC, Mnist, SVHN, Cifar10, and Cifar100.

## References

[1] Zhang J., Wang W., Lu C., Wang J., Sangaiah A. K. (2020). Lightweight deep network for traffic sign classification. *Annals of Telecommunications*.

[2] Stallkamp J., Schlipsing M., Salmen J., Igel C. (2012). Man vs. computer: Benchmarking machine learning algorithms for traffic sign recognition. *Neural Networks*.

[3] Timofte R., Zimmermann K., Van Gool L. (2014). Multi-view traffic sign detection, recognition, and 3d localization. *Machine Vision and Applications*.

[4] Zhe Z., Liang D., Zhang S., Huang X., Hu S. Traffic sign detection and classification in the wild.

[5] He S., Chen L., Zhang S. (2021). Automatic recognition of traffic signs based on visual inspection. *IEEE ACCESS*.

[6] Liu S., Cai T., Tang X., Zhang Y., Wang C. (2022). Visual recognition of traffic signs in natural scenes based on improved RetinaNet. *Entropy*.

[7] Johner F. M., Wassner J. Efficient evolutionary architecture search for cnn optimization on gtsrb.

[8] Qian R., Yue Y., Coenen F., Zhang B. Traffic sign recognition with convolutional neural network based on max pooling positions.

[9] Qiumei Z., Dan T., Fenghua W. (2019). Improved convolutional neural network based on fast exponentially linear unit activation function. *IEEE Access*.

[10] Gu J., Wang Z., Kuen J. (2018). Recent advances in convolutional neural networks. *Pattern Recognition*.

[11] Krizhevsky A., Hinton G. (2009). *Learning Multiple Layers of Features From Tiny Images*.

[12] Deng J., Dong W., Socher R., Li L., Li K., Fei-Fei L. Imagenet: a large-scale hierarchical image database.

[13] Ameur Z., Anis L., Anis S. (2021). A lightweight model for traffic sign classification based on enhanced LeNet-5 network. *Journal of Sensors*.

[14] Ke Z., Yufei Z., Dongmei F. (2021). Learning region-based Attention network for traffic sign recognition. *Sensors*.

[15] Manasa R., Karibasappa K., Kumar S. M. (2021). Differential evolution evolved RBFNN based automated recognition of traffic sign images. *Results in Control and Optimization*.

[16] Cires¸An D., Meier U., Masci J., Schmidhuber J. (2012). Multi-column deep neural network for traffic sign classification. *Neural Networks*.

[17] Szegedy C., Liu W., Jia Y. Going deeper with convolutions.

[18] Simonyan K., Zisserman A. (2014). Very deep convolutional networks for large-scale image recognition. https://arxiv.org/abs/1409.1556.

[19] Arman H. W. A., Arefin S., Shihavuddin A. S. M., Hasan M. A. (2021). DeepThin: a novel lightweight CNN architecture for traffic sign recognition without GPU requirements. *Expert Systems with Applications*.

[20] Cao J., Song C., Peng S., Xiao F., Song S. (2019). Improved traffic sign detection and recognition algorithm for intelligent vehicles. *Sensors*.

[21] Wong A., Shafiee M. J., St Jules M. (2018). Micronnet: a highly compact deep convolutional neural network architecture for real-time embedded traffic sign classification. *IEEE Access*.

[22] Haloi M. (2015). Traffic sign classification using deep inception based convolutional networks. http://arxiv.org/abs/1511.02992.

[23] Arcos-García Á., Álvarez-García J. A., Soria-Morillo L. M. (2018). Deep neural network for traffic sign recognition systems: an analysis of spatial transformers and stochastic optimisation methods. *Neural Networks*.

[24] Ioffe S., Szegedy C. (2015). Batch normalization: Accelerating deep network training by reducing internal covariate shift. http://arXiv.org/abs/1502.03167.

[25] Szegedy C., Vanhoucke V., Ioffe S., Shlens J., Wojna Z. Rethinking the inception architecture for computer vision.

[26] Tabernik D., Skoaj D. (2019). Deep learning for large-scale traffic-sign detection and recognition. *IEEE Transactions on Intelligent Transportation Systems*.

[27] Stallkamp J., Schlipsing M., Salmen J., Igel C. The german traffic sign recognition bench mark: a multi-class classification competition.

[28] Cem B., Tahsin K. (2021). Proper estimation of surface roughness using hybrid intelligence based on artificial neural network and genetic algorithm. *Journal of Manufacturing Processes*.

[29] Nasibeh R. R., Mohammad-R A-T., Alireza A. (2021). Experiment-based affect heuristic using fuzzy rules and Taguchi statistical method for tuning complex systems. *Expert Systems with Applications*.

[30] Balaji K., Siva Kumar M., Yuvaraj N. (2021). Multi objective taguchi–grey relational analysis and krill herd algorithm approaches to investigate the parametric optimization in abrasive water jet drilling of stainless steel. *Applied Soft Computing*.

[31] Zhang L., Zhang M. (2021). Image reconstruction of electrical capacitance tomography based on optimal simulated annealing algorithm using orthogonal test method. *Flow Measurement and Instrumentation*.

[32] Netzer Y., Wang T., Coates A., Bissacco A., Wu B., Andrew Y. Reading digits in natural images with unsupervised feature learning.

[33] Jin J., Fu K., Zhang C. (2014). Traffic sign recognition with hinge loss trained convolutional neural networks. *IEEE Transactions on Intelligent Transportation Systems*.

